# Treatment Effects of Upper Limb Action Observation Therapy and Mirror Therapy on Rehabilitation Outcomes after Subacute Stroke: A Pilot Study

**DOI:** 10.1155/2020/6250524

**Published:** 2020-01-02

**Authors:** Yu-Wei Hsieh, Yu-Hsuan Lin, Jun-Ding Zhu, Ching-Yi Wu, Yun-Ping Lin, Chih-Chi Chen

**Affiliations:** ^1^Department of Occupational Therapy and Graduate Institute of Behavioral Sciences, College of Medicine, Chang Gung University, Taoyuan, Taiwan; ^2^Healthy Aging Research Center, Chang Gung University, Taoyuan, Taiwan; ^3^Department of Physical Medicine and Rehabilitation, Chang Gung Memorial Hospital, Linkou, Taiwan; ^4^Department of Physical Medicine and Rehabilitation, Cathay General Hospital, Taipei, Taiwan; ^5^Division of Occupational Therapy, Department of Physical Medicine and Rehabilitation, Taipei Hospital, Ministry of Health and Welfare, New Taipei City, Taiwan; ^6^School of Medicine, College of Medicine, Chang Gung University, Taoyuan, Taiwan

## Abstract

**Background:**

Action observation therapy and mirror therapy, two promising rehabilitation strategies, are aimed at enhancing the motor learning and functional improvement of stroke patients through different patterns of visual feedback and observation.

**Objective:**

This study investigated and compared the treatment effects of the action observation therapy, mirror therapy, and active control intervention on motor and functional outcomes of stroke patients.

**Methods:**

Twenty-one patients with subacute stroke were recruited in this study. All patients were randomly assigned to the action observation therapy, mirror therapy, or active control intervention for 3 weeks. Outcome measures were conducted at baseline, immediately after treatment, and at 3-month follow-up. The primary outcome was the Fugl-Meyer Assessment, and secondary outcomes included the Box and Block Test, Functional Independence Measure, and Stroke Impact Scale. Descriptive analyses and the number of patients whose change score achieved minimal clinically important difference were reported.

**Results:**

Both the action observation therapy and active control intervention showed similar improvements on the Fugl-Meyer Assessment, Box and Block Test, and Stroke Impact Scale. Moreover, the action observation therapy had a greater improvement on the Functional Independence Measure than the other 2 groups did. However, the mirror therapy group gained the least improvements on the outcomes.

**Conclusion:**

The preliminary results found that the patients in the action observation therapy and active control intervention groups had comparable benefits, suggesting that the 2 treatments might be used as an alternative to each other. A further large-scale study with at least 20 patients in each group to validate the study findings is needed. This trial is registered with NCT02871700.

## 1. Introduction

Stroke is a common cause of long-term disability among adults worldwide [1]. Most patients with stroke experience varying degrees of dysfunction, and one major dysfunction is upper limb motor impairment [2, 3]. There is a need to develop effective neurorehabilitation interventions to optimize arm function and reduce disability in patients [4, 5]. Action observation therapy and mirror therapy are two prominent approaches targeting stroke motor and functional recovery, and both are supported by neuroscientific foundations [6–9]. However, the relative treatment effects of the upper limb action observation therapy versus mirror therapy in stroke rehabilitation have not been compared.

The action observation therapy is a developing rehabilitation approach based on the role of the mirror neuron system in motor learning [6]. It is a motor-based technique with cognitive strategies concerning stroke motor recovery [6]. The mirror neuron system is activated during both the execution and observation of an action and is the area responsible for the action observation therapy [10, 11]. The action observation therapy helps stroke patients improve motor skills through observing another individual's normal movements and practicing what they have observed. During the action observation therapy, the participants are commonly asked to carefully observe the actions performed by a healthy person in videos (i.e., the observation phase) and then to physically practice the same actions (i.e., the execution phase). Neural reorganization and motor relearning of patients commonly occur in response to different afferent inputs and visual feedback [12]. The action observation therapy is aimed at promoting these processes to improve motor learning and performance in stroke patients. Recent studies have found positive effects of the action observation therapy on improving motor function and daily performance in stroke patients [13–18].

The mirror therapy was initially developed for alleviating phantom limb pain after amputation and has been applied to stroke rehabilitation in the past two decades [19, 20]. The mirror therapy has gained much attention as a rehabilitation strategy to address patients' arm and hand function following a stroke [20–26]. During the mirror therapy, the participants are instructed to watch the reflection of movements of the unaffected arm in a mirror as if it was the affected one. The mirror therapy creates the illusory visual image that the intact arm is the affected arm and is normally moving, so as to enhance the movements of the affected arm. It provides visual and proprioceptive feedback of the intact arm, which may provide a substitute input for absent or reduced proprioceptive feedback from the affected arm [27]. In addition, the mirror therapy might be associated with the mirror neuron system and promote reorganization and functional recovery [25, 28]. A growing number of studies have shown that the mirror therapy could be a beneficial approach for enhancing patients' motor and function after stroke [20, 21, 23–25, 29, 30].

Over the past few years, both the action observation therapy and mirror therapy have been considered to hold great promise to promote the motor learning and functional recovery of stroke patients. The two approaches involve different patterns of motor observation, imitation, and execution, but they share a similar neural basis in the mirror neuron system. In the action observation therapy, patients are commonly asked to observe the actions performed by another person and to execute the same actions, whereas in the mirror therapy, they observe mirror reflections of the unaffected limb's movement as if it was the affected one. Despite the number of recent reports on these two approaches, no studies to date have directly compared the effects of the upper limb action observation therapy and mirror therapy in patients with stroke. This study aimed to investigate the immediate and retained (i.e., at 3-month follow-up) treatment effects of the action observation therapy and mirror therapy on different aspects of clinical outcomes in patients with stroke as compared with a dose-matched active control intervention group (i.e., bilateral arm training).

## 2. Materials and Methods

### 2.1. Research Design and Procedure

This was a three-arm, single-blind, randomized controlled trial. We followed the methods of Shih et al. which is Methods/design of this trial [31]. However, given the limited funding and study resources, we only applied and analysed some secondary outcomes of the published study protocol [31] in this pilot study. Each participant received intervention for 15 training sessions (60 minutes/day, 5 days/week for 3 weeks). Treatment was provided by licensed occupational therapists who were well trained in the treatment protocols. Furthermore, all outcome measures were administered to the patients by the same rater, who was blinded to the group allocation. Measurements were taken at three time points: baseline (T0), immediately after treatment (T1), and 3 months after treatment (T2).

The participants were randomly allocated to 1 of the 3 intervention groups in a 1 : 1 : 1 ratio. The two stratification factors were the severity of the upper limb motor deficits of the patients (Fugl-Meyer Assessment (FMA) score: 20–40 vs. 40–60 [32]) and the side of lesion (right vs. left) to ensure baseline equivalence among the group. The randomization was carried out using an online web-based randomization tool (freely available at http://www.randomizer.org/). For concealment of allocation, the randomization procedure and assignment were managed by an independent research assistant who was not involved in screening or evaluation of the participants.

### 2.2. Participant Selection

**Figure 1:**
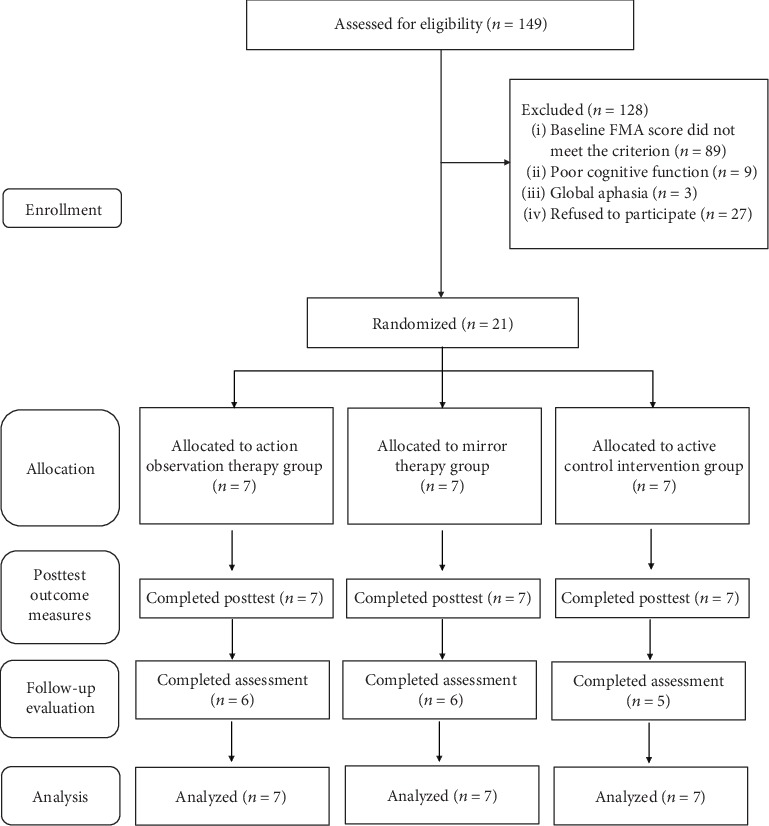
Flowchart of participants.

A total of 21 patients with stroke were recruited in this study (Figure 1). The inclusion criteria of the patients were the following: (1) diagnosis of cerebral ischemic or hemorrhagic stroke, (2) 1 to 6 months since unilateral stroke onset, (3) age between 20 and 80 years, (4) baseline score of the FMA between 20 and 60 [32], (5) ability to follow the study instructions (assessed by the Taiwan version of the Montreal Cognitive Assessment) [33], and (6) ability to participate in study therapy and assessment sessions. The patients were excluded if they had the following: (1) global or receptive aphasia, (2) severe neglect, or (3) major medical problems or comorbidities that influenced the usage of the upper limbs or caused severe pain. All participants provided written informed consent forms approved by the institutional review boards of participating hospitals.

### 2.3. Interventions

**Figure 2:**
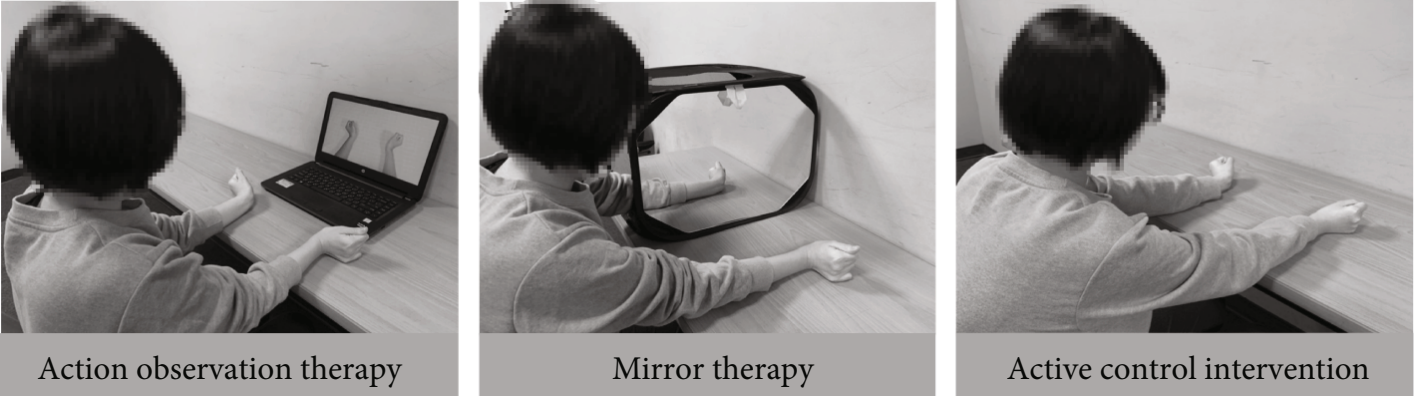
Demonstrations of view of action observation therapy, mirror therapy, and active control intervention during active range of motion exercise.

During treatment, the therapists provided verbal instructions, cues, feedback, and help to patients, when needed. The study intervention (Figure 2) was additional therapy, and all routine conventional rehabilitation programs (e.g., occupational and/or physical therapy) have been provided as usual.

#### 2.3.1. Action Observation Therapy

The patients in the action observation therapy group were required to observe the upper limb movements or functional actions in video clips (i.e., the observation phase) and to execute what they had observed to the best of their ability (i.e., the execution phase). Three common categories of movements and tasks were selected in the action observation therapy protocol based on the related literature and clinical expertise: (a) upper limb active range of motion (AROM) exercises, (b) reaching movement or object manipulation, and (c) upper limb functional tasks. The video movements were displayed from a first-person perspective to make the actions more intuitive and facilitate optimal corticomotor excitability [34]. The actors in the videos were healthy young people. Observing the actions from the first-person perspective means that the observers watch the actions as if seeing through the actor's eyes. It looks like the observers are performing the actions themselves as the same directions and space dimensions of the actors performed.

During phase 1 (10–15 minutes), the patients watched AROM exercises demonstrated in the video clips on a computer screen and executed the observed exercises with both arms and hands simultaneously. In phase 2 (15–20 minutes), the patients were asked to observe one reaching movement or one object manipulation task, depending on the patient's motor ability, for 2 minutes in a video clip, and afterwards to execute the movements that they had observed for 3 minutes. This sequence was repeated 3 times. The reaching movements involved reaching for objects of different sizes and weights at different heights and locations. Object manipulation included in-hand manipulation, grasp and release, and transport and turning objects. Phase 3 (30 minutes) contained one functional task in each session, starting with easy tasks and continuing with increasingly complex tasks. Each functional task was divided into 3 motor acts. For example, the action of cleaning the mouth with a tissue paper was decomposed into the following 3 motor acts: (1) moving hand toward a tissue paper, (2) taking a tissue paper, and (3) bringing the tissue paper toward the mouth and wiping. After observing a motor act in a video clip for 2 minutes, the patients were asked to execute the action they had observed for 3 minutes. For the next 15 minutes, the patients observed the functional task as a complete action for 2 minutes and then executed the entire task for 3 minutes; this sequence was repeated 3 times. Examples of the functional tasks are folding a towel, wiping a table, drinking water, opening a small drawer, and using a mobile phone.

#### 2.3.2. Mirror Therapy

During the mirror therapy, the patients were seated in front of a mirror box placed at their midsagittal plane. The affected arm of the participants was placed inside the mirror box, and the unaffected arm was in front of the mirror. The patient was instructed to watch the mirror reflection of the movement performed by his/her unaffected hand carefully and to imagine that the movement was performed by the affected hand. The participant was also encouraged to move the affected arm and hand as much as they could. In the mirror therapy group, treatment activities also contained AROM exercises (10–15 minutes), reaching movement or object manipulation (15–20 minutes), and functional task practice (30 minutes).

#### 2.3.3. Active Control Intervention—Customary Bilateral Arm Training

The patients in the active control intervention group received dose-matched bilateral arm training provided by a certified occupational therapist, but no video input or mirror box was provided for this group. In the active control intervention, the same 3 categories of movements and tasks as provided in the action observation therapy and mirror therapy groups were used. Treatment programs also included AROM exercises (10–15 minutes), reaching movement or object manipulation (15–20 minutes), and functional task practice (30 minutes). AROM exercises included bilateral shoulder, elbow, forearm, wrist, and finger movements. Object manipulation tasks were in-hand manipulation, grasp and release, and transporting and turning objects with both hands. Examples of functional tasks were reading a magazine, folding clothes, wiping a table, and opening a small drawer with bilateral arm and hand movements. During training, the patients were required to move both of their arms and hands simultaneously as possible. Based on the patient's level of motor ability and progress, the levels of movement and task difficulty could be adjusted accordingly.

### 2.4. Outcome Measures

The patients were assessed at baseline (T0), immediately after treatment (T1), and at 3 months after treatment (T2), and the FMA was used as the primary outcome measure. Secondary outcomes included the Box and Block Test (BBT), Functional Independence Measure (FIM), and Stroke Impact Scale (SIS) version 3.0.

#### 2.4.1. Primary Outcome

The upper limb subscale of the FMA, which has good psychometric properties, is used to assess motor impairments in patients [32, 35]. The 33 items measure the movements, reflexes, and coordination of the upper limb joints. The maximum total score is 66, indicating full upper limb motor recovery. The total score of the FMA can also be separated into the subscores of the proximal shoulder/elbow score (0 to 42) and the distal hand/wrist score (0 to 24). The total, proximal, and distal FMA scores are reported.

#### 2.4.2. Secondary Outcomes

The BBT is a measure of manual dexterity and consists of 150 colored wooden cubes in a box with 2 compartments. The subject uses his/her affected hand to move as many blocks as possible one-by-one from one compartment to the other within 1 minute. The number of blocks transferred is counted as the BBT score. Its reliability and validity are satisfactory in stroke patients [36, 37].

The FIM is a commonly used scale for assessing the performance in basic daily activities. It contains 18 items (i.e., 13 motor and 5 cognition items), and the total score ranges from 0 to 126 [38]. A higher score indicates greater independence in daily activities. The total score and motor score of the FIM are reported. The FIM has good reliability, validity, and responsiveness [39, 40].

The SIS 3.0 is a stroke-specific, patient-reported questionnaire for evaluating the function, participation, and health-related quality of life of stroke survivors. The SIS 3.0 has sound psychometric properties [41]. It consists of 59 items, and higher scores indicate better function and greater participation. The total SIS score and the average score of 4 physical functional domains of the SIS are reported in this study. In addition, an item assessing general perception of recovery since stroke onset on a visual analogue scale of 0 to 100 was also used.

### 2.5. Data Analysis

Baseline characteristics and outcomes of patients were compared among the 3 groups. This study applied an intention-to-treat analysis. For missing data, the last observation carried forward method was used. Descriptive analyses with change scores from T0 to T1 and from T0 to T2 on each outcome were performed. Further, the number of patients whose change score achieved minimal clinically important difference (MCID) on the clinical outcomes in each intervention group was reported. The MCID value was adopted for the established value of the outcome or was set as 10% of the maximum score while it has not been established. Thus, the MCID values of the FMA total, BBT, FIM total, and SIS total scores were 6, 5, 22, and 10 points, respectively [39, 42, 43]. In addition, two-way repeated measures ANOVA was used to evaluate the treatment effects among the 3 treatment groups at 3 time points of assessment. The results of inferential statistics were reported in Supplementary Table 1. Statistical analyses were performed in SPSS 19.0 (SPSS Inc., Chicago, Illinois).

## 3. Results

The baseline demographic and clinical data are summarized in Table 1. There were no statistically significant differences among the 3 groups except for the stroke type, indicating that most of the baseline characteristics among the 3 groups were comparable. In addition, *no adverse effect was reported* by the participants.

### 3.1. Primary Outcome

Table 2 shows that the mean changes in the FMA total scores in the action observation therapy, mirror therapy, and active control intervention groups were 5.14, 2.57, and 7.14 from T0 to T1 and 4.43, 4.71, and 9.86 from T0 to T2, respectively. The mean changes in the FMA proximal scores in the action observation therapy, mirror therapy, and active control intervention groups were 1.71, 1.29, and 3 from T0 to T1 and 2.14, 3.14, and 5 from T0 to T2, respectively. Additionally, the mean changes in FMA distal scores in the action observation therapy, mirror therapy, and active control intervention groups were 3.43, 1.29, and 4.14 from T0 to T1 and 2.29, 1.57, and 4.86 from T0 to T2, respectively (Table 2).

In addition, there were 4, 1, and 5 patients achieving the MCID of the FMA total score after receiving the action observation therapy, mirror therapy, and active control intervention, respectively. Based on the descriptive and MCID results, the patients had similar improvements on the FMA after receiving the action observation therapy and active control intervention. The mirror therapy group gained the least improvement on the FMA among the 3 groups. Most of the improvements were maintained for 3 months.

### 3.2. Secondary Outcomes

The change scores of the BBT in the action observation therapy, mirror therapy, and active control intervention groups were 6.14, 3.43, and 5.14 from T0 to T1 and 8.86, 5, and 8.71 from T0 to T2, respectively (Table 2). In addition, there were 4, 2, and 4 patients achieving the MCID of the BBT score after receiving the action observation therapy, mirror therapy, and active control intervention, respectively.

The mean changes in the FIM total scores in the action observation therapy, mirror therapy, and active control intervention groups were 7.43, 2.14, and 2.57 from T0 to T1 and 12.86, 5.86, and 6.14 from T0 to T2, respectively (Table 2). In addition, there was 1 patient achieving the MCID of the FIM total score after the action observation therapy, but no patient reached the MCID of the FIM total score after the mirror therapy and active control intervention.

Furthermore, the mean changes in the SIS total scores in the action observation therapy, mirror therapy, and active control intervention groups were 8.3, 5.67, and 8.41 from T0 to T1 and 12.83, 13.83, and 9.27 from T0 to T2, respectively (Table 2). In addition, there were 4, 2, and 4 patients achieving the MCID of the SIS total score after receiving the action observation therapy, mirror therapy, and active control intervention, respectively.

According to the descriptive and MCID results on secondary outcomes, both the action observation therapy and active control intervention had similar improvements on the BBT and SIS scores. The action observation therapy group had a greater improvement on the FIM total score than the other 2 groups did. In addition, the patients in the mirror therapy group had the least improvements in most outcomes compared with the other 2 groups.

## 4. Discussion

This is the first study to examine and compare the treatment effects of the upper limb action observation therapy and mirror therapy on different aspects of rehabilitation outcomes in patients with subacute stroke. Based on the descriptive and MCID results, we found that both the action observation therapy and active control intervention showed similar degrees of improvements on the FMA, BBT, and SIS scores. In addition, patients in the action observation therapy group improved most on the FIM score among the 3 groups. However, the mirror therapy group showed the least improvements on the outcomes compared with the other 2 groups. During the period immediately after treatment and at 3-month follow-up, the scores on most outcomes had been maintained in the 3 groups, indicating retention effects. Our preliminary findings showed that the patients in the action observation therapy and active control intervention groups exhibited comparable improvements on most outcomes, but the patients in the mirror therapy group gained the least improvements after treatment.

The action observation therapy and active control intervention (i.e., bilateral arm training) had comparable effects on most rehabilitation outcomes assessed in this study. The patients improved their motor impairment, manual dexterity, and quality of life after receiving the action observation therapy and active control intervention. One explanation for the similar improvements might be the active control intervention used in this study. Our active control intervention was a type of bilateral arm training. However, most previous studies used sham action observation therapy as the control group [13, 17, 18]. In the sham action observation therapy, only static images or pictures were provided during the observation phase, which may have given prominence to the experimental action observation therapy group. In the present study, as compared with the dose-matched active control intervention (i.e., bilateral arm training), the action observation therapy group still gained comparable improvements, suggesting that the 2 treatments might be used as an alternative to each other.

The action observation therapy group (observation plus execution) spent less time and effort on physical practice and execution than the active control intervention and mirror therapy groups because some of the therapy time in the action observation therapy group was the period of observation only. This difference indicates that the action observation therapy might be a suitable alternative for patients with poor physical endurance or those who have difficulty engaging in mirror imagination to improve their function. Nevertheless, the action observation therapy requires much preparation. Video clips need to be made and prepared before the action observation therapy is conducted, and video content must be added or modified in line with the patients' progress and needs. By contrast, the active control intervention is a relatively simple and convenient rehabilitation approach with easier access than the action observation therapy. Each treatment has its own advantages and limitations, and therapists can select the most appropriate one for their specific patients in particular practice settings.

We also found that the patients gained the least improvements after receiving the mirror therapy among the 3 intervention groups. There are some possible reasons for explaining the fewest gains from the mirror therapy. Firstly, the patients were instructed to perform movements and tasks within a mirror box during the mirror therapy. There were limitations while executing movements in the limited space of a mirror box, such as allowing only a small range of motion and limited types of activities. Secondly, the variability and range of motor deficits of patients (i.e., FMA baseline score) in the mirror therapy group were higher than those in the other 2 groups which may also affect its efficacy in this study. Thirdly, some previous studies also showed that the mirror therapy did not lead to better outcomes compared with control interventions [44, 45]. Probably, the conflicts between vision and proprioception caused by not exactly the same movements between the mirror reflection of nonaffected hand and the actual affected hand during the mirror therapy may lead to unpleasant sensations and reduce its treatment effect [44, 46].

We found that greater mean change scores and a higher number of patients achieving the MCID values on the motor and functional outcomes were in the action observation therapy group than in the mirror therapy group. In one previous study of lower limb stroke rehabilitation, the action observation therapy group significantly improved static balance and gait function of patients, and the mirror therapy improved gait function only [47]. One possible reason may be that the motor learning process of action observation and imitation during the action observation therapy might be more intuitive and straightforward than that during the mirror therapy. Motor understanding and learning might be easier for patients with stroke to imitate and learn via video observation than via mirror visual feedback. However, the preliminary results and the mechanisms underlying action observation therapy- and mirror therapy-induced clinical improvements will require further elaboration.

In this pilot study, 85.9% of the patients screened were excluded. The major reason was that the baseline FMA scores of the patients were too high or low (i.e., <20 or >60). A further study should add different types of participating sites or hospitals, and it should also recruit participants from additional sites with higher percentages of patients with moderate to mild motor impairments (i.e., FMA scores between 20 and 60). In addition, the therapy contents and programs of the control intervention might need to be further revised. This pilot study used bilateral arm training, a known and effective treatment, as an active control intervention. However, the design of applying an active control intervention is more suitable for noninferiority clinical trials [48]. Thus, a conventional rehabilitation or usual care group might be more suitable as a control group than is an active comparative group in further research with purposes similar to those of this pilot study.

The limitations of this study need to be addressed. First, this pilot study might not have sufficient total sample size to detect significant interaction effects or group main effects. Future studies should endeavor to recruit larger sample sizes to compare and verify the treatment effects among the 3 groups. About 20 subjects in each group (a total of 60) will be required to have enough power to detect statistical significance in a further study (power = 80%, effect size *f* = 0.42, and *α* = 0.05). Second, we did not select patients with particular stroke types or lesion sites, which may have increased the variability among the patients in this pilot study. Whether the stroke types or different lesion sites of patients (e.g., mirror neurons affected or not) influence the treatment efficacy of the action observation therapy or mirror therapy will need to be investigated in the future. In addition, early studies found that the initial motor function of stroke patients was a significant predictor or a critical determinant of motor recovery after receiving rehabilitation therapies [49–51]. Whether the baseline severity of the upper limb motor deficits of the patients affects the treatment efficacy of the action observation therapy and mirror therapy needs to be further investigated.

## 5. Conclusions

In conclusion, both the action observation therapy and active control intervention had similar degrees of improvements on most outcomes. The mirror therapy group gained the least improvements among the 3 groups. Based on the preliminary study findings, the action observation therapy is a promising alternative to the contemporary intervention of bilateral arm training (i.e., active control intervention) in patients with subacute stroke. Further large-scale studies to examine the clinical efficacy and neural mechanisms of the action observation therapy and mirror therapy in patients with stroke are suggested.

## Figures and Tables

**Table 1:** Baseline characteristics of the participants.

	Action observation therapy (*n* = 7)	Mirror therapy (*n* = 7)	Active control intervention (*n* = 7)	*P*
Gender (male/female)	6/1	6/1	6/1	1.0
Age (years) (mean (SD))	52.77 (11.25)	46.41 (13.45)	54.30 (13.61)	0.49
Onset (months) (mean (SD))	2.86 (1.77)	4.86 (1.95)	2.57 (1.72)	0.06
Lesion side (right/left)	2/5	4/3	3/4	0.85
Stroke type (hemorrhagic/ischemic)	4/3	7/0	2/5	0.03
Education (years) (mean (SD))	12.29 (2.87)	11.86 (2.27)	11.43 (4.12)	0.88
MoCA score (mean (SD))	24.57 (5.29)	25.29 (3.25)	24.00 (5.51)	0.88
FMA score (mean (SD))	42.29 (11.03)	43.29 (13.72)	39.57 (6.55)	0.80

Abbreviations: FMA: Fugl-Meyer Assessment; MoCA: Taiwanese version of Montreal Cognitive Assessment.

**Table 2:** Descriptive statistics for the primary and secondary outcomes.

Variables	Group	Assessment time			Change score	
		T0	T1	T2	T1-T0	T2-T0
FMA-total	Action observation therapy	42.29 (11.03)	47.43 (13.38)	46.71 (11.91)	5.14 (5.27)	4.43 (8.06)
	Mirror therapy	43.29 (13.72)	45.86 (14.66)	48.00 (15.20)	2.57 (3.82)	4.71 (4.50)
	Active control intervention	39.57 (6.55)	46.71 (8.44)	49.43 (7.93)	7.14 (3.29)	9.86 (4.45)

FMA-proximal	Action observation therapy	30.86 (5.15)	32.57 (6.40)	33.00 (5.89)	1.71 (3.15)	2.14 (3.53)
	Mirror therapy	30.57 (5.97)	31.86 (5.87)	33.71 (6.37)	1.29 (3.25)	3.14 (4.26)
	Active control intervention	30.86 (3.67)	33.86 (4.56)	35.86 (4.53)	3.00 (2.52)	5.00 (3.46)

FMA-distal	Action observation therapy	11.43 (7.66)	14.86 (8.59)	13.71 (6.95)	3.43 (2.51)	2.29 (4.89)
	Mirror therapy	12.71 (8.77)	14.00 (9.50)	14.29 (9.69)	1.29 (1.60)	1.57 (1.90)
	Active control intervention	8.71 (5.28)	12.86 (5.15)	13.57 (5.29)	4.14 (2.04)	4.86 (2.79)

BBT	Action observation therapy	9.86 (11.39)	16.00 (11.14)	18.71 (10.08)	6.14 (5.98)	8.86 (7.29)
	Mirror therapy	17.28 (17.50)	20.71 (16.94)	22.29 (18.18)	3.43 (3.55)	5.00 (6.06)
	Active control intervention	9.00 (7.66)	14.14 (11.61)	17.71 (14.43)	5.14 (4.22)	8.71 (7.76)

FIM-total	Action observation therapy	103.86 (22.61)	111.29 (11.54)	116.71 (10.87)	7.43 (13.16)	12.86 (17.71)
	Mirror therapy	110.14 (5.34)	112.29 (5.06)	116.00 (4.69)	2.14 (0.90)	5.86 (3.80)
	Active control intervention	113.57 (5.47)	116.14 (4.85)	119.71 (4.50)	2.57 (1.90)	6.14 (4.81)

FIM-motor	Action observation therapy	74.00 (16.45)	79.71 (8.73)	84.29 (6.85)	5.71 (8.69)	10.29 (13.43)
	Mirror therapy	78.43 (6.05)	80.57 (5.35)	83.86 (3.34)	2.14 (0.90)	5.43 (3.51)
	Active control intervention	82.00 (5.26)	84.00 (4.00)	86.00 (3.65)	2.00 (1.53)	4.00 (4.12)

SIS-total	Action observation therapy	64.00 (13.28)	72.31 (11.75)	76.83 (10.43)	8.30 (5.09)	12.83 (10.44)
	Mirror therapy	62.96 (9.93)	68.63 (7.44)	76.79 (6.25)	5.67 (7.01)	13.83 (10.59)
	Active control intervention	68.85 (10.28)	77.26 (8.71)	78.12 (8.97)	8.41 (7.20)	9.27 (9.12)

SIS-physical function	Action observation therapy	53.37 (13.73)	65.85 (9.77)	72.12 (11.52)	12.49 (9.16)	18.75 (17.84)
	Mirror therapy	56.07 (14.05)	63.96 (14.07)	74.40 (13.83)	7.90 (7.68)	18.33 (11.27)
	Active control intervention	62.03 (11.71)	74.29 (11.34)	74.69 (12.09)	12.26 (9.02)	12.66 (8.15)

SIS-recovery	Action observation therapy	51.43 (17.73)	58.57 (10.69)	58.57 (11.07)	7.14 (12.54)	7.14 (15.77)
	Mirror therapy	49.29 (15.92)	57.14 (14.68)	63.57 (11.07)	7.86 (3.93)	14.29 (8.38)
	Active control intervention	61.43 (12.15)	70.71 (15.92)	65.71 (17.18)	9.29 (10.18)	4.29 (5.35)

Abbreviations: FMA: Fugl-Meyer Assessment; BBT: Box and Block Test; FIM: Functional Independence Measure; SIS: Stroke Impact Scale. Note: T0: baseline; T1: immediately after treatment; T2: 3 months after treatment.

## Data Availability

Data are available from the corresponding authors on reasonable request.

## Supplementary Materials

Supplementary Materials Supplementary Table 1: inferential statistics for the primary and secondary outcomes.
