# Supplementary material for: Treatment Effects of Upper Limb Action Observation Therapy and Mirror Therapy on Rehabilitation Outcomes after Subacute Stroke: A Pilot Study
Source: Behav Neurol. 2020 Jan 2;2020:6250524. doi: 10.1155/2020/6250524 (PMC7199557; doi:10.1155/2020/6250524)
Supplement: Supplementary Materials — Supplementary Table 1: inferential statistics for the primary and secondary outcomes. [file 6250524.f1.docx]

**Supplementary Table 1.** Inferential statistics for the primary and secondary outcomes

| Variables | Time | Group | | | Interaction effect | | Main effect | | | |
| --- | --- | --- | --- | --- | --- | --- | --- | --- | --- | --- |
|  |  | Action observation therapy | Mirror therapy | Active control intervention | Group*Time | | Group | | Time | |
|  |  |  |  |  | *F* | *P* | *F* | *P* | *F* | *P* |
| FMA-total | Pretest | 42.29 (11.03) | 43.29 (13.72) | 39.57 (6.55) | 1.82 | 0.146 | 0.003 | 0.997 | 20.234 | < 0.001* |
|  | Posttest | 47.43 (13.38) | 45.86 (14.66) | 46.71 (8.44) |  |  |  |  |  |  |
|  | Follow-up | 46.71 (11.91) | 48.00 (15.20) | 49.43 (7.93) |  |  |  |  |  |  |
| FMA-proximal | Pretest | 30.86 (5.15) | 30.57 (5.97) | 30.86 (3.67) | 0.911 | 0.449 | 0.180 | 0.837 | 13.317 | < 0.001* |
|  | Posttest | 32.57 (6.40) | 31.86 (5.87) | 33.86 (4.56) |  |  |  |  |  |  |
|  | Follow-up | 33.00 (5.89) | 33.71 (6.37) | 35.86 (4.53) |  |  |  |  |  |  |
| FMA-distal | Pretest | 11.43 (7.66) | 12.71 (8.77) | 8.71 (5.28) | 1.81 | 0.148 | 0.137 | 0.873 | 15.162 | < 0.001* |
|  | Posttest | 14.86 (8.59) | 14.00 (9.50) | 12.86 (5.15) |  |  |  |  |  |  |
|  | Follow-up | 13.71 (6.95) | 14.29 (9.69) | 13.57 (5.29) |  |  |  |  |  |  |
| BBT | Pretest | 9.86 (11.39) | 17.28 (17.50) | 9.00 (7.66) |  |  |  |  |  |  |
|  | Posttest | 16.00 (11.14) | 20.71 (16.94) | 14.14 (11.61) | 0.584 | 0.623 | 0.468 | 0.634 | 19.971 | < 0.001* |
|  | Follow-up | 18.71 (10.08) | 22.29 (18.18) | 17.71 (14.43) |  |  |  |  |  |  |
| FIM-total | Pretest | 103.86 (22.61) | 110.14 (5.34) | 113.57 (5.47) | 0.905 | 0.432 | 0.771 | 0.477 | 11.018 | 0.002* |
|  | Posttest | 111.29 (11.54) | 112.29 (5.06) | 116.14 (4.85) |  |  |  |  |  |  |
|  | Follow-up | 116.71 (10.87) | 116.00 (4.69) | 119.71 (4.50) |  |  |  |  |  |  |
| FIM-motor | Pretest | 74.00 (16.45) | 78.43 (6.05) | 82.00 (5.26) | 1.060 | 0.372 | 0.852 | 0.443 | 12.166 | 0.002* |
|  | Posttest | 79.71 (8.73) | 80.57 (5.35) | 84.00 (4.00) |  |  |  |  |  |  |
|  | Follow-up | 84.29 (6.85) | 83.86 (3.34) | 86.00 (3.65) |  |  |  |  |  |  |
| SIS-total | Pretest | 64.00 (13.28) | 62.96 (9.93) | 68.85 (10.28) | 0.837 | 0.476 | 0.661 | 0.528 | 25.677 | < 0.001* |
|  | Posttest | 72.31 (11.75) | 68.63 (7.44) | 77.26 (8.71) |  |  |  |  |  |  |
|  | Follow-up | 76.83 (10.43) | 76.79 (6.25) | 78.12 (8.97) |  |  |  |  |  |  |
| SIS-physical function | Pretest | 53.37 (13.73) | 56.07 (14.05) | 62.03 (11.71) | 0.995 | 0.406 | 0.707 | 0.506 | 29.070 | < 0.001* |
|  | Posttest | 65.85 (9.77) | 63.96 (14.07) | 74.29 (11.34) |  |  |  |  |  |  |
|  | Follow-up | 72.12 (11.52) | 74.40 (13.83) | 74.69 (12.09) |  |  |  |  |  |  |
| SIS-recovery | Pretest | 51.43 (17.73) | 49.29 (15.92) | 61.43 (12.15) | 1.418 | 0.248 | 1.239 | 0.313 | 9.785 | < 0.001* |
|  | Posttest | 58.57 (10.69) | 57.14 (14.68) | 70.71 (15.92) |  |  |  |  |  |  |
|  | Follow-up | 58.57 (11.07) | 63.57 (11.07) | 65.71 (17.18) |  |  |  |  |  |  |

Abbreviations: FMA, Fugl-Meyer Assessment; BBT, Box and Block Test; FIM, Functional Independence Measure; SIS, Stroke Impact Scale.
